# Job Crafting and Nurses' Presenteeism: The Effects of Job Embeddedness and Job Irreplaceability

**DOI:** 10.3389/fpubh.2022.930083

**Published:** 2022-06-28

**Authors:** Taotao Liu, Wei Wang, Geyan Shan, Yijie Zhang, Jie Liu, Yongxin Li

**Affiliations:** ^1^Institute of Psychology and Behavior, Faculty of Education, Henan University, Kaifeng, China; ^2^Huaihe Hospital of Henan University, Henan University, Kaifeng, China

**Keywords:** presenteeism, job crafting, job embeddedness, job irreplaceability, nurse

## Abstract

**Background:**

Presenteeism is defined as the behavior of people who insist on attending work despite complaints of ill health that should prompt rest and absence from work. Due to the heavy workloads and irreplaceable duties of the nursing service, nurses are a typical representative group suffering from presenteeism. Although more scholars have recently begun focusing on presenteeism, an abundant number of studies have tended to focus on presenteeism's external objective factors. There is, thus, a lack of studies based on variables related to the intra-individual initiative. This study aimed to address this gap by exploring the relationship between job crafting and nurses' presenteeism from the perspective of the individual internal initiative. Furthermore, this study also aimed to examine job embeddedness' mediating effect and job irreplaceability's moderating effect on presenteeism.

**Methods:**

A total of 900 nurses from a 3A-graded hospital in Henan Province were invited to participate in the online study in October, November, and December 2021, respectively. Participants were asked to complete Self-report scales on job crafting, job embeddedness, job irreplaceability, and presenteeism at three time points above. Job crafting was measured at Time 1, job embeddedness and job irreplaceability were measured at Time 2, and presenteeism was measured at Time 3.

**Results:**

Presenteeism was significantly associated with differences in participants' age and tenure. Job crafting was significantly positively associated with job embeddedness, and job embeddedness was significantly negatively correlated with presenteeism. Job embeddedness mediated the relationship between job crafting and presenteeism. Job irreplaceability moderated the relationship between job embeddedness and presenteeism.

**Conclusions:**

This study explored job crafting's influence mechanism on nurses' presenteeism, which is beneficial to providing effective suggestions for managing and preventing the incidence of nurses' presenteeism. Future research should consider expanding the sampling area and enriching the occupational fields of included participants to conduct a more in-depth discussion on the relationship between job crafting and nurses' presenteeism.

## Introduction

Presenteeism is defined as the behavior of people who insist on attending work despite complaints of ill health that should prompt rest and absence from work (1). Compared to other occupational groups, nurses face a heavier workload, higher work irreplaceability, and poorer health status (2). Therefore, they are often regarded by scholars as a group with a high incidence of presenteeism (3). Previous studies indicate that 82.08% of nurses have worked while sick from the perspective of the direct leader of nurses, and the proportion of nurses who Self-reported experiencing presenteeism was as high as 94.25% (4). With the COVID-19 pandemic, healthcare workers experience unprecedented challenges (5). It presented exceeding workloads for healthcare workers, which not only increased nurses' work stress and exhaustion (6, 7) but also caused delays and avoidance of medical care worldwide (8, 9). Under such circumstances, nurses' physical and mental health was damaged (6), and the phenomenon of nurses working with ill health also increased to a great extent.

From the personal health perspective, the recovery theory indicated that individuals with ill health need certain resources to recover, such as temporary rest or staying away from work (10). Presenteeism deprives individuals' opportunity to recover from stress and illness, not only reducing the acquisition of recovery resources but causing further deterioration of health conditions and long-term damage to physical and mental health. Empirical studies also showed that nurses' presenteeism can cause cumulated fatigue and stress, lead to impaired physical and mental health (11–13), and further intensify their job burnout (14), Self-depletion (15), and depersonalization symptoms (16). From the social aspect, monetary losses are also experienced by the healthcare organization due to presenteeism (17). Shan et al. (4) found that there were ¥4.38 billion and ¥2.88 billion in annual losses, respectively, according to nurses' and chief nurses' presenteeism reports. Therefore, to avoid a series of negative outcomes, it is necessary to focus on preventing and reducing the occurrence of nurse presenteeism.

Most previous studies have explored factors related to the aspects of work, such as leadership, colleagues, and organizations, to combat presenteeism (18–20). These are objective and stable, seldom involving factors related to personal initiative. With the coming of the digital network and intelligence era, human initiative plays an increasingly important role in social development and construction, which can prompt individuals to actively seek out work resources to effectively cope with potential work pressures and demands. This active resource-seeking behavior is called “job crafting” in the organizational management field. Specifically, job crafting is defined as the behavior of employees who spontaneously and proactively adjust job requirements and resources to achieve a better person-job fit, thereby increasing the meaning and experience of work (21).

According to the Job Demands-Resources Model (22), the resources that employees obtained through job crafting may help individuals to cope with job demands. Yi and Kim (23) pointed out that job crafting as an initiative behavior is a key factor influencing presenteeism. However, the relationship between job crafting and presenteeism still needs to be clarified through further empirical research. Only one Danish scholar has explained the motivation behind people's choice of presenteeism from the perspective of job crafting and suggested the necessity of exploring the relationship between job crafting and presenteeism (24). Hence, this study aimed to address this gap and further investigate job crafting's influence mechanism on nurses' presenteeism. It is conducive to explaining the occurrence mechanism of nurses' presenteeism so that effective suggestions could be provided for preventing nurses' presenteeism based on it. It also contributes to investigating the relationship between job crafting and negative organizational behavior (presenteeism), which can enrich the research on the outcome variables of job crafting.

In the healthcare industry, nurses with a high job crafting level may actively communicate and collaborate with others to seek more resources and support, which may deepen their connection with others and embeddedness in the organization, thus, creating a strong sense of belonging (25). High organization embeddedness may promote pro-organizational behaviors, promoting unwillingness to damage the organization's interests. Presenteeism behavior has always been regarded as a negative organizational behavior due to the loss of organizational performance in the long run (26). Consequently, to maintain the organization's interests, nurses with high embeddedness may avoid actions with the potential to damage organizational performance, such as presenteeism. Based on this view, job crafting may reduce presenteeism by increasing the level of job embeddedness.

Existing research indicates that job demand is a vital antecedent for presenteeism (23). High job irreplaceability is usually associated with heavy work demands, such as high difficulty and high technical requirements, which will put pressure on nurses (27), cause more health problems (2), and thus increase presenteeism behavior. Nurses with high job embeddedness are associated with more social and skill resources (28), which can effectively address high job irreplaceability's negative impact, thereby reducing the incidence of presenteeism. Conversely, for low job embeddedness, due to the lack of effective resources, nurses' stress might be increased when faced with high job irreplaceability, resulting in more health risks and presenteeism behaviors (29).

**Figure 1:**
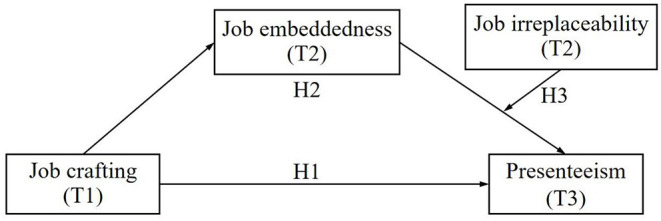
Hypothesized conceptual model.

In summary, this study built a moderated mediation model to explore the relationship mechanism between job crafting and nurses' presenteeism. Specifically, this study examined the mediating role of job embeddedness on the relationship between job crafting and nurses' presenteeism and further explored the moderating role of job irreplaceability between job embeddedness and nurses' presenteeism. The hypothesized model is shown in Figure 1.

## Theory and Hypotheses

### Job Crafting and Presenteeism

When individuals are sub-healthy, additional physical and psychological resources are required to face the heavy and complex nursing work. Furthermore, when nurses must work whilst unhealthy, prime resources would be consumed, resulting in a shortage of overall coping resources. If the lack of overall resources is not adequately restored in time, an imbalance in various bodily functions may result. If this situation continues, the individual's physical and mental health will be impacted to a greater extent (30).

Job crafting is defined as employees' behavior in actively changing their job design and perceptions to improve their sense of work meaning and maintain a better person-job fit (21). Leana et al. (31) highlighted that nurses would proactively search for a range of support resources from their manager and organization in the process of job crafting through various ways, such as organizational training, benefit programs, and leadership or colleague information sharing. According to the Conservation of Resources (COR) theory and Job Demands-Resources Model (JDR) (22), individuals seek to protect and promote their resources, such as objects, conditions, personal characteristics, energy, etc. (32). Perception of resource loss and threat to resources can result in stress responses (33). Oppositely, abundant work resources could assist nurses in coping with potential work demands, relieve work pressure, and reduce the consumption of physical and mental resources, thereby reducing the occurrence of presenteeism. Simultaneously, nurses could gain more capital and reinforced support from work resources to address the loss of performance or other adverse impacts caused by absence from work, so that if ill health occurs, they may tend to choose sick leave instead of presenteeism (28, 34). Therefore, we put forward the following hypothesis:

***Hypothesis 1**: Job crafting has a negative effect on nurses*' *presenteeism*.

### Mediation Effects of Job Embeddedness

Job crafting is a process meant to obtain more resources with which individuals can actively change their job design and their own cognition (21). If nurses obtained rich work resources through job crafting, their work enthusiasm would be stimulated, and work happiness would be improved (35). Nurses would then be more willing to integrate into the organization and take action to increase their degree of embeddedness in their work. Job embeddedness refers to the degree to which employees are embedded in the work and social network (e.g., organizations and communities) (36). The more embedded in the organization and social network individuals are, the more tightly connected with the organization and social network they will be, and the stronger attachment they would form to the organization. Halbesleben and Wheeler (25) indicate that individuals' strong sense of attachment to their organization, and a strong sense of belonging, would make them unwilling to damage the organization's interests and thus enact Pro-organizational behaviors. Therefore, nurses would try to avoid presenteeism due to its tendency to cause long-term organizational performance loss (26). To summarize, the resources that nurses gain from job crafting would increase their level of job embeddedness, which further makes nurses reluctant to presenteeism because of the detrimental effect on organizational performance. From this, we come up with the following hypothesis:

***Hypothesis 2**: Job embeddedness has an indirect effect on the relationship between job crafting and nurses*' *presenteeism*.

### Moderation Effects of Job Irreplaceability

Job irreplaceability is the extent to which the job content cannot be replaced by others. High job irreplaceability may result due to understaffing, lack of resources, and the task's specificity that prevents one's work from being substituted by another (37). High job irreplaceability is often associated with high job demands, which can lead to higher stress for nurses (27), and cause more health problems (2), thus increasing presenteeism. Previous studies have shown that job irreplaceability can positively affect presenteeism (38). Considering that nurses with high job embeddedness tend to have a stronger attachment to the organization (39), and are associated with more social and skill resources (28), they could address work-related problems more effectively and cope with the work pressure and potential work demand caused by high irreplaceability. Thus, reducing the presenteeism caused by job irreplaceability. In contrast, nurses with low job embeddedness have a weaker sense of attachment to the organization, often lacking sufficient resources to cope with the heavy workload, and potential work pressure, brought about by job irreplaceability (29), further intensifying job irreplaceability's impact on nurse's presenteeism. Thus, under high job irreplaceability, nurses with poor job embeddedness are more likely to experience a series of health problems and presenteeism behavior. Given that low job irreplaceability among nurses tends to be associated with more reasonable solutions to cope with absence, these individuals could more effectively cope with job challenges when relying on existing physical and mental resources, thereby less presenteeism would appear among nurses, even during poor job embeddedness. Hence, we hypothesize the following:

***Hypothesis 3**: Job irreplaceability moderates the relationship between job embeddedness and nurses*' *presenteeism*.

## Materials and Methods

### Participants

This study utilized convenience sampling. Participants were recruited from a 3A-graded hospital in the Henan Province, China. Before the investigation, all participants were informed about the research's purpose, relevant precautions for filling out the questionnaire, and the principle of confidentiality. After obtaining their consent and cooperation, online data was collected through “www.wjx.cn” in October, November, and December 2021, respectively. Job crafting was measured in October (Time1), job embeddedness and job irreplaceability were measured in November (Time 2), and presenteeism was measured in December (Time 3). At these three time points, 900 questionnaires were distributed. After deleting invalid questionnaires at Time 1, 844 questionnaires were obtained with an effective response rate of 93.78%. After deleting invalid questionnaires at Time 2, 738 questionnaires were obtained with an effective response rate of 82%. At Time 3, 739 questionnaires were obtained after deleting invalid questionnaires, and the effective response rate is 82.11 %. Then, we screened and excluded the invalid questionnaires; the exclusion criteria were: (1) repeated questionnaires; (2) mobile phone numbers that could not be matched; (3) scores of the three times responses were the same or regular. Finally, we obtained 490 valid matching questionnaires. In this study, 316 (64.5%) participants were 31 years old and above while 174 (35.5%) were 30 years old and below; 364 (74.3%) participants had tenure of 6 years or more while 126 (25.7%) participants had tenure of 5 years or less; 456 (93.1%) participants had Bachelor and above degrees while 34 (6.9%) had Junior college and below degree. Meanwhile, the G^*^power was used to calculate the minimum sample size needed for the hypothesized model. The effect size *f*^2^ was set at 0.15, the significant level (α) was set at 0.05, and the power at 0.95, and the number of predictors 5. The result showed that 138 samples were needed to validate the hypotheses of this study. Around 490 samples that enrolled in our study met the requirement of sample size for data analysis.

### Measures

#### Job Crafting

Job Crafting was assessed by a 21-item measure by Tims et al. (40), translated by Lou (41), and widely used in China. The scale contains four subscales, namely increasing social job resources (five items; such as “I ask colleagues for advice”), increasing structural job resources (five items; such as “I try to develop myself professionally”), increasing challenging job demands (five items; such as “I try to make my work more challenging by examining the underlying relationships between aspects of my job”), and decreasing hindering job demands (six items; such as “I manage my work so that I try to minimize contact with people whose problems affect me emotionally”). 21 items were measured on a Likert 5-point scale ranging from 1 (strongly disagree) to 5 (strongly agree). For this scale, Cronbach's alpha Co-efficient was 0.96.

#### Job Embeddedness

Job Embeddedness was assessed by part of the Job Embeddedness Scale, developed by Mitchell et al. (36), and translated by Wang (42). A total of 14 typical items were selected from the 40-item scale to evaluate nurses' job embeddedness. Three subscales were comprised of the scale, namely organizational links (three items; such as “I keep close relationships with colleagues at work”), organizational fit (five items; such as “I feel like I'm a good fit for my current job”), and organizational sacrifice (six items; such as “Resignation will cause a lot of damage to my family and me”). 14 items were measured on a Likert 5-point scale ranging from 1 (strongly disagree) to 5 (strongly agree). For this scale, Cronbach's alpha Co-efficient was 0.94.

#### Presenteeism

The 11-item Nurse Presenteeism Questionnaire (NPQ) developed by Shan et al. (43) was employed to measure nurses' presenteeism behavior. Items included: “Although you felt dizzy or had a headache, you still persevered in going to work.” Eleven items were measured on a Likert 4-point scale: 0 (never), 1 (once), 2 (2~5 times), and 3 (more than five times), with high scores representing more frequent instances of presenteeism. For this questionnaire, Cronbach's alpha Co-efficient was 0.95.

#### Job Irreplaceability

Job irreplaceability was assessed by the single item scale, as used by Aronsson and Gustafsson (37) in their research: “If you are absent from work for up to a week, what proportion of your tasks must you take up again on your return?” Responses on a Likert 4-point scale: 1 (none or only a small proportion), 2 (somewhat less than half), 3 (somewhat more than half), and 4 (virtually all). The higher scores reflect a higher irreplaceability level of the participants.

### Data Analysis

SPSS, AMOS22.0, and the PROCESS (39) plug-in were used to test hypotheses. Specifically, it includes four steps. First, SPSS and AMOS were used to test for common method bias. Secondly, a descriptive analysis of participants' presenteeism scores was used. Third, the Pearson correlation analysis was evaluated to test the correlations between variables. Finally, the postulated hypotheses were tested by PROCESS and hierarchical linear regression.

### Ethics Statement

The study design was approved by the Henan University, and the involving human participants were reviewed and approved by The Ethical Review Board of the Institution of Psychology and Behavior. The participants provided their written informed consent to participate in this study.

## Results

### Tables' Preliminary Analysis

#### Missing Data Analysis

The 844 valid questionnaires from the first survey and the 490 matching questionnaires from Time 3 were analyzed. First, a total of 354 missing sample data were screened out by subtracting 490 matched questionnaires from the 844 total questionnaires at Time 1, the result of which was coded as the “Missing Sample” group. Then, the 490 valid questionnaires were coded as the “Effective Sample” group. We compared the differences in job crafting scores and demographic variables between the two groups, respectively. Results indicated that the score difference between missing samples and valid samples on job crafting was not significant (*P* > 0.05). There was no significant difference in tenure and education level between the two groups (*P*_*s*_ > 0.05). However, there was a significant difference in age (*P* < 0.05). Specifically, in the Effective Sample, 316 (64.5%) participants were 31 years old and above while 174 (35.5%) were not. However, in the Missing Sample, 199 (56.2%) participants were 31 years old and above while 155 (43.8%) were not. Overall, the subsequent analysis would not be affected seriously by these missing data.

#### Common Method Bias

Considering that the questionnaires used in this study are all Self-reporting scales, common method bias was analyzed in diverse ways. Primarily, the Pre-control was conducted, collecting data from different time waves to reduce common method bias. Then, Harman's univariate analysis was used after collecting data through SPSS 26. These results indicated that there were seven factors with eigenvalues greater than 1 and that the first factor explains 34.43 % of the variance. Thus, no serious common method bias appeared in the study. Furthermore, the common method bias was tested through AMOS 22.0 by controlling for the effects of an unmeasured latent method. First, Model 1 was constructed through confirmatory factor analysis. Second, Model 2 was constructed including the method factor. Third, the main fit indices of Model 1 and Model 2 were compared. Results showed that the changes of each fitting index were all less than 0.04 (ΔRMSEA = 0.003, ΔCFI = 0.015, ΔIFI = 0.015, ΔNFI = 0.016, ΔTLI = 0.011) and that the model was not significantly changed after adding the common method factor, which further indicated that no serious common method bias was present in the study.

### The Scores of NPQ and the Differences in Demographic Characteristics

Table 1 presents the descriptive statistics of the NPQ scores. Results indicated a significant difference in NPQ scores (*t* = −5.31 *P* < 0.01) relating to nurses' age. The NPQ scores of participants aged 31 and above were significantly higher than that of those aged 30 and below, additionally, nurses with varying tenure also had significant differences in NPQ scores (*t* = −5.97, *P* < 0.01). Specifically, NPQ scores were significantly higher in nurses with 6 years or more of tenure than in nurses with 5 years or less.

**Table 1:** Descriptions and correlations among demographic characteristics and NPQ scores (*n* = 490).

**Variables**	**Categories**	**Case**	***x* ±*s***	***t***	***P*-value**
Age	≤ 30	174	2.63 ± 0.86	−5.31	0.000
	≥31	316	3.04 ± 0.79		
Tenure	≤ 5	126	2.53 ± 0.86	−5.97	0.000
	≥6	364	3.02 ± 0.79		
Education	Junior college and below	34	3.11 ± 0.70	1.61	0.109
level	Bachelor and above	456	2.88 ± 0.84		

*NPQ, nurse presenteeism questionnaire*.

### Variables Correlations

The results of the correlated analysis were shown in Table 2. Specifically, job crafting had a significantly positive relationship with job embeddedness (*r* = 0.54, *P* < 0.01), and job embeddedness was significantly negatively correlated with presenteeism (*r* = −0.11, *P* < 0.05). However, job crafting was not significantly related to presenteeism (*r* = −0.02, *P* > 0.05), which indicated that Hypothesis 1 was not supported.

**Table 2:** Correlation of research variables (*n* = 490).

**Variables**		**M ±SD**	**1**	**2**	**3**	**4**	**5**	**6**
1	Age	1.64 ± 0.48	1					
2	Tenure	1.74 ± 0.44	0.76**	1				
3	JCS	4.18 ± 0.62	−0.05	−0.03	1			
4	JEQ	3.70 ± 0.71	−0.05	–0.11*	0.54**	1		
5	NPQ	2.90 ± 0.83	0.23**	0.26**	−0.02	–0.11*	1	
6	JIQ	3.06 ± 0.98	−0.02	0.01	0.12**	0.11*	0.08	1

Age: 1 = ≤ 30; 2 = ≥31. Tenure: 1 = ≤ 5; 2 = ≥6. JCS, job crafting scale; JEQ, job embeddedness questionnaire; NPQ, nurse presenteeism questionnaire. JIQ, job irreplaceability questionnaire. *P < 0.05; **P < 0.01.

Considering that the statistical power would decline by an excess of control variables (44), age and tenure have a significant influence on nurses' presenteeism in this study, while education level has no significant effect on it; thus, nurses' age and tenure were included as control variables in the follow-up analysis and education level was not included.

### Mediation Effects of Job Embeddedness

Model 4 in the pluggable unit of PROCESS in SPSS compiled by Hayes (45) was used to test job embeddedness' mediating effect on the relationship between job crafting and presenteeism while controlling for age and tenure. The results were presented in Table 3.

**Table 3:** Results of the mediation effect of job embeddedness (*n* = 490).

**Variables**	**Presenteeism**		**Job embeddedness**		**Presenteeism**	
	**β**	***t***	**β**	***t***	**β**	***t***
Age	0.14	1.21	0.17	2.01*	0.17	1.41
Tenure	0.38	2.92**	−0.29	–3.07**	0.34	2.61**
Job crafting	−0.01	−0.18	0.61	14.23**	0.07	1.02
Job					−0.13	–2.15*
embeddedness				
*R^2^*	0.07		0.30		0.08
*F*	12.36**		70.89**		10.49**

*P < 0.05; **P < 0.01.

Results showed that the direct effect of job crafting on presenteeism was not significant (β = −0.01, *P* > 0.05). Further, the mediating effect of job embeddedness was analyzed, which indicated that the 95 % bias-corrected confidence interval for the indirect effect of job embeddedness excluded zero [CI = (−0.161, −0.003)] and the mediator effect was −0.081. After controlling the mediation variable of job embeddedness, job crafting's direct effect on presenteeism was not significant [95 % CI = (−0.066, 0.208), *t* = 1.018, *P* > 0.05]. Therefore, job embeddedness played a complete mediation role between job crafting and presenteeism, and Hypothesis 2 was supported.

### Moderation Effects of Job Irreplaceability

Model 1 in the pluggable unit of PROCESS was used to test the moderating effect of job irreplaceability on job embeddedness and presenteeism while controlling for age and tenure. The results showed that the main effects of job embeddedness (β = −0.07, *P* < 0.05) and job irreplaceability (β = 0.08, *P* < 0.05) on presenteeism were significant, and that the interaction of the two also significantly impacts presenteeism (β = −0.07, *P* < 0.05). The results provided evidence that job irreplaceability has a significant moderation effect on the relationship between job embeddedness and presenteeism. Hypothesis 3 was thus confirmed.

**Figure 2:**
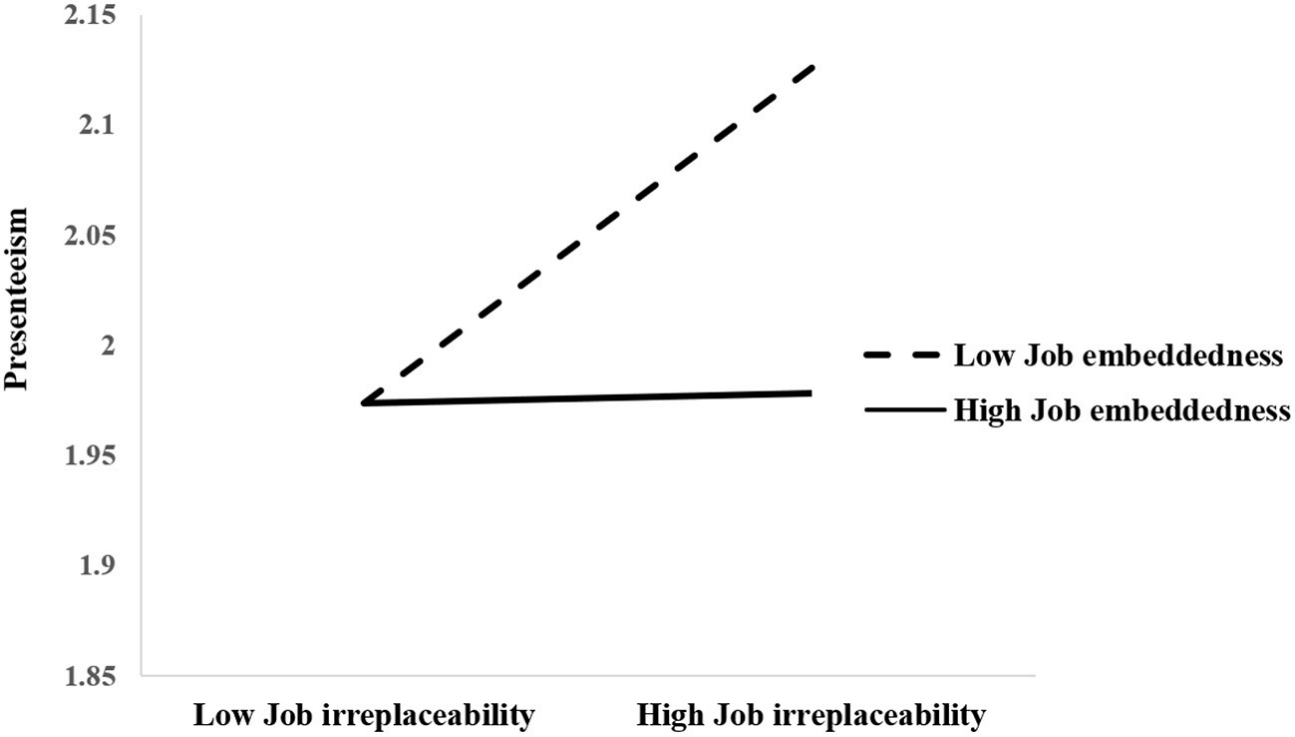
Moderation effect of job irreplaceability between job embeddedness and presenteeism.

Then, a simple slope test was used to reveal job irreplaceability's moderating trend on the relationship between job embeddedness and presenteeism (see Figure 2). As indicated in Figure 2, in the condition of low job irreplaceability, job embeddedness had no significant impact on presenteeism (β = −0.001, *P* > 0.05). While in the high job irreplaceability group, the impact of job embeddedness on presenteeism was significant (β = −0.15, *P* < 0.01). With increasing job irreplaceability, nurses with lower job embeddedness suffered more presenteeism than the nurses with higher job embeddedness.

**Figure 3:**
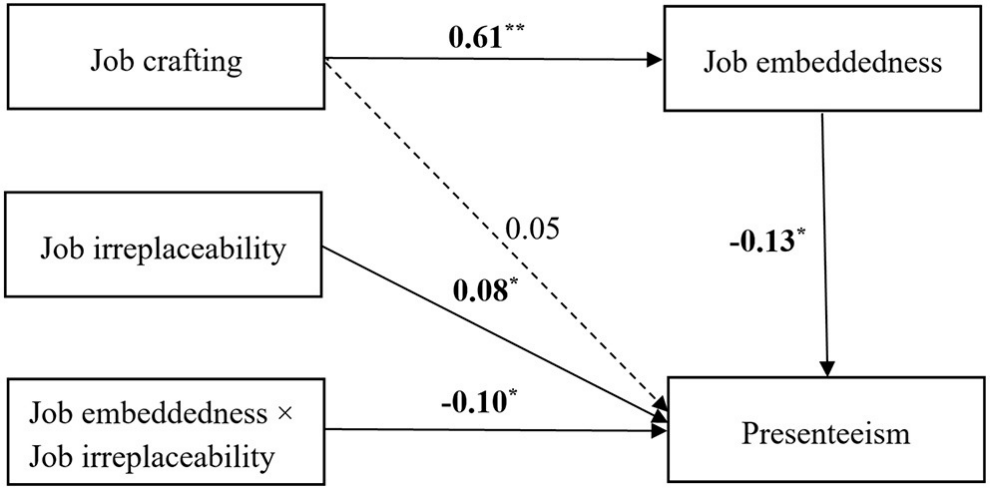
Results of the moderated mediation model.

Furthermore, the moderated mediation model test was conducted through Model 14 in the pluggable unit of PROCESS in SPSS. The results are presented in Table 4 and Figure 3. The moderated mediation effect is significant with an index of−0.63, and a 95 % bias-corrected confidence interval of [−0.122, −0.001]. Specifically, when the level of job irreplaceability was low (−1SD), the mediating effect of the mediator was not significant and the effect was 0.016 [95 % CI = (−0.123, 0.084)]. When the level of job irreplaceability was high (+1SD), the mediating effect of job embeddedness was significant and the effect was −0.137 [95 % CI = (−0.228, −0.051)].

**Table 4:** Test of the moderated mediation model (*n* = 490).

**Variable**	**Job embeddedness**		**Presenteeism**	
	**β**	***t***	**β**	***t***
Age	0.17	2.01*	0.17	1.41
Tenure	−0.29	–3.07**	0.33	2.56*
Job crafting	0.61	14.23**	0.05	0.69
Job embeddedness			−0.13	–2.08*
Job irreplaceability			0.08	2.05*
Job embeddedness ×			−0.10	–2.08*
Job irreplaceability			
*R^2^*	0.30		0.10
*F*	70.89**		8.46**

*P < 0.05; **P < 0.01.

## Discussion

### Discussion of Results

Under the global epidemic of COVID-19, as the vanguard of epidemic prevention and control, healthcare workers are fighting against the high risk of disease transmission and facing high health threats; thus, their health issues are worth more attention than ever before. Previous research has shown that the COVID-19 pandemic had varied degrees of detrimental impact on the mental health of healthcare workers (46). For example, healthcare workers experienced higher levels of depression, anxiety (6), and emotional exhaustion than usual (47). Meanwhile, the presenteeism of healthcare workers also increased during this period (48). Given that nurses' presenteeism tends to lead to more work errors (49) and increased patient health and safety risks (50), paying attention to nurses' working health behavior is vital for promoting nurses' level of healthcare service. This study investigated the relationship mechanism of job crafting and nurses' presenteeism through the perspective of internal motivation, which enriches the research regarding presenteeism's occurrence mechanism. Moreover, the present study explored job embeddedness' mediating role and job irreplaceability's moderating role in the relationship between job embeddedness and presenteeism, which blends the factors of personal motivation and work characteristics in an integrated model. The results may thus trigger more contemplations regarding the prevention and management of nurses' presenteeism.

First, the results indicated an association between older age and greater levels of presenteeism, which is consistent with previous studies (23). This may be due to younger nurses' tendency to face the status of volatile income security and lower organizational loyalty, which makes them consider their work as less important than their health, and therefore, be inclined to take sick leave when they are in poor health (51). Meanwhile, the results also showed an association for nurses between longer tenure and greater presenteeism, which is consistent with previous studies (4). This may be due to nurses with longer tenure regarding their presenteeism as a role model for their colleagues with shorter tenure (52), however, nurses with longer tenure also closely interact with patients in daily work. They may thus be afraid that their absence would affect the patient's recovery and are thus inclined to presenteeism under poor health instead of absence (29).

Second, the results revealed that job crafting can reduce presenteeism by increasing job embeddedness. Leana et al. (31) highlighted that in the process of job crafting, nurses would proactively search for a range of support resources from their manager and organization. These resources included organizational training, benefit programs, and leadership or colleague information sharing, which aids the improvement of nurses' sense of work meaning and job control, enhancing the degree of job fit with abilities, working styles, and hobbies (53), thus to increase the level of job embeddedness. Nurses with a high level of job embeddedness tend to attach great importance to organizational interests, keep high organizational loyalty (39), and aim to maximize benefits for the organization. Hence, they might be keenly aware of the dark side of presenteeism, a negative work state that would lead to depletion of work capacity and loss of organizational productivity (54), and thus, avoid its occurrence. In general, individuals' job crafting would increase their job embeddedness, which would result in having higher organizational loyalty and tighter organizational ties, and thus, further influence them to avoid conducting presenteeism to maintain organizational interests. In previous studies on job crafting, scholars mainly examined the relationship between job crafting and positive outcome variables. Although a scholar mentioned that job crafting may be related to presenteeism (negative variable) and presenteeism should be explained from the perspective of job crafting (24), there is still a lack of relevant research. This study provides an empirical basis for this by exploring the relationship between job crafting and presenteeism, which can expand the research scope of outcome variables related to job crafting. Meanwhile, this study expanded the research scope of the antecedent variable of presenteeism, which shifts the perspective from negative variables possibly related to presenteeism to the positive variable (job crafting) that could play a role in presenteeism.

Third, the findings confirmed that job irreplaceability played a moderating role in the relationship between job embeddedness and presenteeism. The high job irreplaceability indicates a lack of work resources, requiring more complex and special skills, which often require nurses to devote more time and energy to complete the work and thus deplete more physical and psychological resources. Nurses with high job embeddedness tend to have a stronger sense of attachment to the organization (39). In this situation, they are associated with more social and skill resources (28), which can help them effectively cope with the potential job demands and job stress caused by high irreplaceability, thus reducing the risk of presenteeism. Therefore, high job irreplaceability may not be a key factor affecting nurses with high job embeddedness's decision to choose presenteeism. On the contrary, people with low job embeddedness lack effective resources to deal with the potential stress and demands caused by high job irreplaceability, so they need to consume additional resources. When such physical and mental resources are excessively consumed, and not replenished in time, exhaustion and burnout will occur, which in turn increases presenteeism (29). Therefore, high job irreplaceability increases the incidence of presenteeism in people with low job embeddedness. For low job irreplaceability, work tasks and responsibilities are simpler, work pressure is lower, and nurses can effectively cope with it through existing resources. Therefore, the impact of low job irreplaceability on nurses with different levels of embeddedness is not significant.

### Theoretical Implications

Based on the COR and JDR theories, this study explored the mechanism of job crafting on nurses' presenteeism, providing the empirical basis for COR and JDR theories. Job crafting and presenteeism belong to two independent fields in previous studies. Although one scholar suggested that presenteeism should be explored from the perspective of job crafting (24), there is still a lack of relevant empirical research. This study establishes a bridge between job crafting and presenteeism as it is the first to examine the relationship between job crafting and nurses' presenteeism, as well as introduce job embeddedness and job irreplaceability. It not only draws the research on the antecedent variables of presenteeism into a new perspective but also enriches the research scope of the consequences variables of job crafting.

First, numerous studies focused on the negative influence of presenteeism on individual physical health, mental health, and organizational productivity (55). Research on its antecedent variables mostly focused on external objective targets such as colleagues, leaders, and organizations (20), and less attention was paid to whether factors related to individual internal motivation can affect presenteeism. Based on the perspective of individual internal motivation, this study explored the impact of job crafting on nurses' presenteeism, which is conducive to enriching and improving the research on presenteeism.

Second, job crafting, as a top-down behavior, is regarded as a positive behavior of employees' spontaneous initiative, and the related research has always focused on its positive outcomes, such as employee job satisfaction (21), job happiness (56, 57), and organizational performance (58). In this study, it is indicated that unhealthy working behavior (presenteeism) could be indirectly decreased through job crafting, which is helpful in enriching research on job crafting's inhibiting effect on negative outcomes and broadening the job crafting research perspective.

### Practical Implications

There are two valuable practical implications in the present study. First, we explored the effect of job crafting on nurses' presenteeism and examined the mediating effect of job embeddedness and the moderating effect of job irreplaceability between them. This study highlights the importance of caring about healthcare practitioners, which might increase the attention of the healthcare industry to presenteeism, thus arousing concern for nurses' physical and mental health. The findings help formulate corresponding management systems effectively and reasonably, which could provide nurses with a good support environment and adequate job replacement resources, from the perspective of job crafting, for alleviating the occurrence of presenteeism.

Second, in the field of organizational management, most of the research on job crafting involved its positive outcome factors. By examining the relationship between job crafting and the negative variables-nurses' presenteeism, the study reveals that job crafting can reduce the occurrence of nurses' presenteeism through increasing job embeddedness. It is, thus, beneficial for organizational management departments, and individuals, to focus on job crafting's preventive effect on presenteeism, which could promote the full positive role of job crafting behaviors.

### Limitations and Future Research

Our study has several notable limitations. Our data was collected during a period when the epidemic situation in China was generally stable, indicating there were no confirmed COVID-19 positive cases in our sample area, and the work arrangement of the nursing professionals was close to the normal before the epidemic. However, the international epidemic is not stable, and domestic coastal cities, such as Shanghai and Guangzhou, have imported cases with the positive nucleic acid test for COVID-19. Therefore, the item description of presenteeism may trigger associations about COVID-19, leading to a certain degree of bias in the Self-reports of presenteeism behaviors among nurses. Furthermore, Self-reported scales were the main data measurement tools used in this study. When answering questions, social expectations and self-approval might thus influence the objectivity of the final data. Multi-angle measurement methods should be adopted in future research, such as adding situational imagination and experimental operations, or combining the evaluation of leaders and colleagues, to improve the objectivity and scientific nature of the research.

Due to the particularity of the nursing group, differences exist from other groups regarding occupational stability, gender ratios, and work pressure. Therefore, future research can be conducted on groups of different occupations, which will further broaden the related research on presenteeism behavior. Additionally, this study focuses on a large 3A-graded hospital in a prefecture-level city in the Henan Province. The sample size and number are, thus, representative, however, there are differences in the rules, regulations, and economic income of hospital nurses in different regions. Future research can thus collect data from nurses in different regions, such as general hospitals, or hospitals in other provinces and rural areas, further comparing and refining the research on nurses' presenteeism behavior.

## Conclusion

Our study investigated the impact of job crafting on presenteeism from the internal motivation perspective and examined the mediation effect of job embeddedness and the moderation effect of job irreplaceability. Results showed that although job crafting, a traditionally positive variable, and presenteeism, a traditionally negative variable, were not significantly correlated, job crafting could indirectly reduce the occurrence of presenteeism by increasing job embeddedness. Additionally, job irreplaceability played a moderating role in the relationship between job embeddedness and presenteeism. The predictive effect of job embeddedness on presenteeism was stronger with the improvement of job irreplaceability. The results, thus, enrich the research on the antecedent variables of presenteeism, fill the gap in the research on the proactive antecedence related to presenteeism, and may further expand research in occupational health psychology, organizational management psychology, and other fields. Therefore, hospital management should pay attention to providing nurses with appropriate and rich resource support, as well as adding specialized staffing shifts and replacement positions. Nurses should also engage in more job crafting behaviors to exploit more resource support and strengthen organizational ties to reduce presenteeism.

## Data Availability Statement

The raw data supporting the conclusions of this article will be made available by the authors, without undue reservation.

## Ethics Statement

The studies involving human participants were reviewed and approved by Research Ethics Committee of the Institute of Psychology and Behavior, Henan University. The patients/participants provided their written informed consent to participate in this study.

## Author Contributions

Under the direction of WW, YL, TL, and GS generated the idea and designed the study, they were the principal investigators for the study and was the primary writer of the manuscript. YZ and JL supported the data input, data analysis, and data collection. All authors were involved in developing, editing, reviewing, and providing feedback for this manuscript and have given approval for the final version to be published.

## Funding

This study was supported by the National Natural Science Foundation of China (Grant No. 72101083).

## Conflict of Interest

The authors declare that the research was conducted in the absence of any commercial or financial relationships that could be construed as a potential conflict of interest.

## Publisher's Note

All claims expressed in this article are solely those of the authors and do not necessarily represent those of their affiliated organizations, or those of the publisher, the editors and the reviewers. Any product that may be evaluated in this article, or claim that may be made by its manufacturer, is not guaranteed or endorsed by the publisher.

## Abbreviations

NPQ: nurse presenteeism questionnaire.

## References

[1] Aronsson G Gustafsson K Dallner M. Sick but yet at work. An empirical study of sickness presenteeism. J Epidemiol Community Health. (2000) 54:502–9. 10.1136/jech.54.7.502 10846192 PMC1731716

[2] Mitchell KJ Vayalumkal JV. Sickness presenteeism: the prevalence of coming to work while ill among pediatric resident physicians in Canada. Paediatr Child Health. (2017) 22:84–8. 10.1093/pch/pxx026 29479187 PMC5804915

[3] Gustafsson K Marklund S. Consequences of sickness presence and sickness absence on health and work ability: a Swedish prospective cohort study. Int J Occup Med Environ Health. (2011) 24:153–65. 10.2478/s13382-011-0013-3 21526385

[4] Shan G Wang S Wang W Guo S Li Y. Presenteeism in nurses: prevalence, consequences, and causes from the perspectives of nurses and chief nurses. Front Psychiatry. (2020) 11:584040. 10.3389/fpsyt.2020.584040 33488418 PMC7819974

[5] Ehrlich H Mckenney M Elkbuli A. Protecting our healthcare workers during the COVID-19 pandemic. Am J Emerg Med. (2020) 38:1527–8. 10.1016/j.ajem.2020.04.024 32336585 PMC7162741

[6] Leng M Xiu H Yu P Feng J Wei Y Cui Y. Current state and influencing factors of nurse resilience and perceived job-related stressors. J Contin Educ Nurs. (2020) 51:132–7. 10.3928/00220124-20200216-08 32119108

[7] Lorente L Vera M Peiró T. Nurses stressors and psychological distress during the COVID pandemic: the mediating role of coping and resilience. J Adv Nurs. (2020) 77:1335–44. 10.1111/jan.14695 33210768 PMC7753515

[8] Lupuoru I Ciobanu D Ursaru M Blan GG Grigorovici A. Difficulties in treating a patient with multiple cancers in the COVID-19 pandemic. Chirurgia. (2020) 5:115. 10.21614/chirurgia.115.5.670 33138905

[9] Nikolayevskyy V Holicka Y Soolingen DV Werf M Cirillo D. Impact of covid-19 pandemic on tuberculosis laboratory services in Europe. Eur Respir J. (2020) 57:2003890. 10.1183/13993003.03890-2020 33184119 PMC7670866

[10] Meijman TF Mulder G. Psychological aspects of workload. In: Drenth PJD, Thierry H, de Wolff CJ, editors. Handbook of Work and Organizational: Work Psychology. London: Psychology Press/Erlbaum (UK) Taylor & Francis (1998). p. 5–33.

[11] Taloyan M Aronsson G Leineweber C Magnusson Hanson LM Alexanderson K Westerlund H. Sickness presenteeism predicts suboptimal self-rated health and sickness absence: a nationally representative study of the Swedish working population. PLoS ONE. (2012) 7:e44721. 10.1371/journal.pone.0044721 22984547 PMC3439368

[12] Fiorini LA Houdmont J Griffiths A. Nurses' perceived work performance and health during presenteeism: cross-sectional associations with personal and organizational factors. J Nurs Manag. (2020) 13065. 10.1111/jonm.13065. [Epub ahead of print]. 32506664

[13] Skagen K Collins AM. The consequences of sickness presenteeism on health and wellbeing over time: a systematic review. Soc Sci Med. (2016) 161:169–77. 10.1016/j.socscimed.2016.06.005 27310723

[14] Yildirim MH Saygin M Uguz S. Effects of presenteeism syndrome on employees' burnout levels. Int J Soc Sci. (2014) 6:1–10.

[15] Rivkin W Diestel S Gerpott FH Unger D. Should I stay or should I go? The role of daily presenteeism as an adaptive response to perform at work despite somatic complaints for employee effectiveness. J Occup Health Psychol. (2022). 10.1037/ocp0000322. [Epub ahead of print]. 35298208

[16] Demerouti E Le Blanc PM Bakker AB Schaufeli WB Hox J. Present but sick: a three-wave study on job demands, presenteeism and burnout. Career Dev Int. (2009) 14:50–68. 10.1108/13620430910933574

[17] Howard JT Howard KJ. The effect of perceived stress on absenteeism and presenteeism in public school teachers. J Workplace Behav Health. (2020) 35:100–16. 10.1080/15555240.2020.1724794

[18] Reuter M Wahrendorf M Di Tecco C Probst TM Ruhle S Ghezzi V. Do temporary workers more often decide to work while sick? Evidence for the link between employment contract and presenteeism in Europe. Int J Environ Res Public Health. (2019) 16:1868. 10.3390/ijerph16101868 31137850 PMC6572370

[19] Dietz C Zacher H Scheel T Otto K Rigotti T. Leaders as role models: effects of leader presenteeism on employee presenteeism and sick leave. Work Stress. (2020) 34:300–22. 10.1080/02678373.2020.1728420

[20] Liu B Lu Q Zhao Y Zhan J. Can the psychosocial safety climate reduce ill-health presenteeism? Evidence from Chinese healthcare staff under a dual information processing path lens. Int J Environ Res Public Health. (2020) 17:1–17. 10.3390/ijerph17082969 32344791 PMC7215888

[21] Tims M Bakker AB. Job crafting: toward a new model of individual job redesign. SA J Ind Psychol. (2010) 36:1–9. 10.4102/sajip.v36i2.841

[22] Bakker AB Demerouti E. The job demands-resources model: state of the art. J Manag Psychol. (2007) 22:309–28. 10.1108/0268394071073311531861812

[23] Yi JS Kim H. Factors related to presenteeism among South Korean workers exposed to workplace psychological adverse social behavior. Int J Environ Res Public Health. (2020) 17:3472. 10.3390/ijerph17103472 32429315 PMC7277895

[24] Giæver F Løvseth Lise T. Exploring presenteeism among hospital physicians through the perspective of job crafting. Qual Res Organ Manag Int J. (2019) 15:296–314. 10.1108/QROM-11-2018-1699

[25] Halbesleben JRB Wheeler AR. The relative roles of engagement and embeddedness in predicting job performance and intention to leave. Work Stress. (2008) 22:242–56. 10.1080/02678370802383962

[26] Letvak SA Ruhm CJ Gupta SN. Nurses' presenteeism and its effects on Self-reported quality of care and costs. Am J Nurs. (2012) 112:30–8; quiz 48, 39. 10.1097/01.NAJ.0000411176.15696.f9 22261652

[27] Caverley N Cunningham JB Macgregor JN. Sickness presenteeism, sickness absenteeism, and health following restructuring in a public service organization. J Manag Stud. (2007) 44:304–19. 10.1111/j.1467-6486.2007.00690.x

[28] Tabak F Hendy NT. Work engagement: trust as a mediator of the impact of organizational job embeddedness and perceived organizational support. Organ Manag J. (2016) 13:21–31. 10.1080/15416518.2015.1116968

[29] Mckevitt C Morgan M Dundas R Holland WW. Sickness absence and “working through” illness: a comparison of two professional groups. J Public Health Med. (1997) 19:295–300. 10.1093/oxfordjournals.pubmed.a024633 9347453

[30] Lu LL Cooper C Yen Lin H. A cross-cultural examination of presenteeism and supervisory support Career. Dev Int. (2013) 18:440–56. 10.1108/CDI-03-2013-0031

[31] Leana C Appelbaum E Shevchuk I. Work process and quality of care in early childhood education: the role of job crafting. Acad Manag J. (2009) 52:1169–92. 10.5465/amj.2009.47084651

[32] Halbesleben JR Harvey J Bolino MC. Too engaged? A conservation of resources view of the relationship between work engagement and work interference with family. J Appl Psychol. (2009) 94:1452–65. 10.1037/a0017595 19916655

[33] Hobfoll SE. Conservation of resources: a new attempt at conceptualizing stress. Am Psychol. (1989) 44:513–24. 10.1037/0003-066X.44.3.513 2648906

[34] Zhang L Fan C Deng Y Lam CF Hu E Wang L. Exploring the interpersonal determinants of job embeddedness and voluntary turnover: a conservation of resources perspective. Hum Resour Manag J. (2019) 29:413–32. 10.1111/1748-8583.12235

[35] Kim M Beehr TA. Can empowering leaders affect subordinates' wellbeing because they encourage subordinates' job crafting behaviors? J Leadership Organ Stud. (2018) 25:184–96. 10.1177/1548051817727702

[36] Mitchell TR Holtom BC Lee TW Sablynski CJ Erez M. Why people stay: using job embeddedness to predict voluntary turnover. Acad Manag J. (2001) 44:1102–21. 10.5465/3069391

[37] Aronsson G Gustafsson K. Sickness presenteeism: prevalence, attendance-pressure factors, and an outline of a model for research. J Occup Environ Med. (2005) 47:958–66. 10.1097/01.jom.0000177219.75677.17 16155481

[38] Hu WJ. The Influence of Replaceability on Presenteeism—The Adjustment of Position and Gender (Master thesis). Institute of Psychology, Chinese Academy of Sciences (2015).

[39] Kiazad K Holtom BC Hom PW Newman A. Job embeddedness: a multifoci theoretical extension. J Appl Psychol. (2015) 100:641–59. 10.1037/a0038919 25774569

[40] Tims M Bakker AB Derks D. Development and validation of the job crafting scale. J Vocat Behav. (2012) 80:173–86. 10.1016/j.jvb.2011.05.00933512763

[41] Lou TY. A Study on the Impact of Job Insecurity on Job Crafting: A Moderated Mediation Model (Master thesis). Jinan University (2020).

[42] Wang Y. Study on the Influence of Difference Sequence and Job Embedding on the Employee Turnover Intention (Master thesis). Shandong Agricultural University (2020).

[43] Shan G Wang S Feng K Wang W Guo S Li Y. Development and validity of the nurse presenteeism questionnaire. Front Psychol. (2021) 12:679801. 10.3389/fpsyg.2021.679801 34093374 PMC8175652

[44] Becker TE. Potential problems in the statistical control of variables in organizational research: a qualitative analysis with recommendations. Organ Res Methods. (2005) 8:274–89. 10.1177/1094428105278021

[45] Hayes AF. Introduction to mediation, moderation, and conditional process analysis: a regression-based approach. J Educ Meas. (2013) 51:335–7. 10.1111/jedm.12050

[46] Oliveira MMD Treichel CADS Bakolis I Alves PF Coimbra VCC Cavada GP. Mental health of nursing professionals during the COVID-19 pandemic: a cross-sectional study. Rev Saude Publica. (2022) 56:8. 10.11606/s1518-8787.2022056004122 35293941 PMC8910133

[47] Wang H Zhou X Jia X Song C Luo X Zhang H. Emotional exhaustion in front-line healthcare workers during the covid-19 pandemic in wuhan, China: the effects of time pressure, social sharing and cognitive appraisal. BMC Public Health. (2021) 21:829. 10.1186/s12889-021-10891-w 33931034 PMC8085471

[48] White-Means SI Warren CL Osmani AR. The organizational impact of presenteeism among key healthcare workers due to the COVID-19 pandemic. Rev Black Polit Econ. (2022) 49:20–40. 10.1177/00346446211065175 35291319 PMC8914299

[49] Niven K Ciborowska N. The hidden dangers of attending work while unwell: a survey study of presenteeism among pharmacists. Int J Stress Manag. (2015) 22:207–21. 10.1037/a0039131

[50] Freeling M Rainbow JG Chamberlain D. Painting a picture of nurse presenteeism: a multi-country integrative review. Int J Nurs Stud. (2020) 109:103659. 10.1016/j.ijnurstu.2020.103659 32585449

[51] Gustafsson K Marklund S Leineweber C Bergström G Aboagye E Helgesson M. Presenteeism, psychosocial working conditions and work ability among care workers-A cross-sectional Swedish population-based study. Int J Environ Res Public Health. (2020) 17:2419. 10.3390/ijerph17072419 32252368 PMC7177781

[52] Kinman G Clements AJ Hart J. When are you coming back? Presenteeism in UK prison officers. Prison J. (2019) 99:363–83. 10.1177/0032885519838019

[53] Chen CY Yen CH Tsai FC. Job crafting and job engagement: the mediating role of person-job fit. Int J Hosp Manag. (2014) 37:21–8. 10.1016/j.ijhm.2013.10.006

[54] Jiang H Jia H Zhang J Li Y Song F Yu X. Nurses' occupational stress and presenteeism: the mediating role of public service motivation and the moderating role of health. Int J Environ Res Public Health. (2021) 18:3523. 10.3390/ijerph18073523 33805328 PMC8036313

[55] Côté K Lauzier M Stinglhamber F. The relationship between presenteeism and job satisfaction: a mediated moderation model using work engagement and perceived organizational support. Eur Manag J. (2021) 39:270–8. 10.1016/j.emj.2020.09.001

[56] Tims M Bakker AB Derks D. The impact of job crafting on job demands, job resources, and wellbeing. J Occup Health Psychol. (2013) 18:230–40. 10.1037/a0032141 23506549

[57] Tims M Bakker AB Derks D. Examining job crafting from an interpersonal perspective: is employee job crafting related to the wellbeing of colleagues? Appl Psychol. (2015) 64:727–53. 10.1111/apps.12043

[58] Tims M Bakker AB Derks D van Rhenen WV. Job crafting at the team and individual level. Group Organ Manag. (2013) 38:427–54. 10.1177/105960111349242132204448

